# Security-Enhanced Push Button Configuration for Home Smart Control †

**DOI:** 10.3390/s17061334

**Published:** 2017-06-08

**Authors:** Junghee Han, Taejoon Park

**Affiliations:** 1 School of Electronics and Information Engineering, Korea Aerospace University, 76 Hanggongdaehang-ro, Goyang-si, Gyeonggi-do 412-791, Korea; 2 Department of Robotics Engineering, Hanyang University, 55 Hanyangdaehak-ro, Ansan, Gyeonggi-do 15588, Korea; taejoon@hanyang.ac.kr

**Keywords:** smart home, security, push button configuration, Wi-Fi, wireless protected setup

## Abstract

With the emergence of smart and converged home services, the need for the secure and easy interplay of various devices has been increased. Push Button Configuration (PBC) is one of the technologies proposed for easy set-up of a secure session between IT and consumer devices. Although the Wi-Fi Direct specification explicitly states that all devices must support the PBC method, its applicability is very limited. This is because the security vulnerability of PBC can be maliciously exploited so that attackers can make illegitimate sessions with consumer devices. To address this problem, this paper proposes a novel Security-enhanced PBC (SePBC) scheme with which we can uncover suspicious or malicious devices. The proposed mechanism has several unique features. First, we develop a secure handshake distance measurement protocol by preventing an adversary sitting outside the region from maliciously manipulating its distance to be fake. Second, it is compatible with the original Wi-Fi PBC without introducing a brand-new methodology. Finally, SePBC uses lightweight operations without CPU-intensive cryptography computation and employs inexpensive H/W. Moreover, it needs to incur little overhead when there is no attack. This paper also designs and implements the proposed SePBC in the real world. Our experimental results and analysis show that the proposed SePBC scheme effectively defeats attacks on PBC while minimizing the modification of the original PBC equipment.

## 1. Introduction

With the emergence of smart and converged home services, there has been rapid increase in the need for the seamless interplay of devices such as mobile handsets, TVs, set-top boxes, smart meters and all sorts of sensor devices for smart home/building environments. For example, smart phones have become full-featured mobile gateways capable of connecting automatically to other devices or sensors on behalf of the users. This trend inevitably necessitates wireless network connectivity among these devices to provide users with new exciting services such as three-screen play and advanced metering infrastructure for smart grids.

In order to respect such needs, many companies recently adopted various types of Internet of Things (IoT) data link protocols. In particular, Wi-Fi, IEEE 802.15.4 ZigBee and Bluetooth are typical IoT data link protocols, all of which support various smart home-related profiles including home automation and smart energy profiles. Each protocol has its own pros and cons in many aspects. For example, Wi-Fi is known to provide shorter delay (i.e., ~1.5 ms) than ZigBee (i.e., ~20 ms) and Bluetooth (i.e., ~2.5 ms) at the cost of the power consumption of the receiving device [1]. Exploiting these underlying IoT data link protocols, several major IT companies such as Google, Samsung and Apple have developed home automation systems, Weave/Brillo [2], SmartThings [3] and HomeKit [4], respectively. These systems help users to setup and develop smart home applications. In this environment, it is important to offer easy, but secure setup of Wi-Fi, ZigBee and Bluetooth connections among devices to better satisfy the users.

A few industry standards have been recognized to meet this requirement of hassle-free setup. Such standards include WPS (Wi-Fi Protected Setup) by the Wi-Fi Alliance [5]. WPS allows any device to easily setup a secure connection with an Access Point (AP). WPS almost automatically configures the connectivity between them by using a WPA/WPA2 security module coupled with options, such as Push Button Configuration (PBC). With PBC, a user simply pushes buttons on AP and CE devices to securely connect the device to the AP with enabling data encryption.

Another standard is RF4CE (Radio Frequency for Consumer Electronics). It was originally developed by major CE companies, such as Panasonic and Samsung Electronics, which later merged with the ZigBee Alliance [6]. ZigBee RF4CE targets primarily at radio frequency (RF) remote controls for devices using a simplified version of the ZigBee protocol optimized for CE devices. RF4CE also supports the PBC mode for a user to press buttons twice to set up a secure pairing between ZigBee-equipped devices.

As such, easy and simple pairing methods among IoT devices play a key role in the smart home environment with Wi-Fi WPS, ZigBee RF4CE and Bluetooth [7] networks. It enables users to easily setup a secure session between devices with minimal interactions with themselves. In particular, each PBC-speaking device is equipped with a button to be pressed by a user to execute PBC. Hence, if a user wants to make a pairing between two devices using PBC, he only needs to press each of the buttons. It thus relieves him/her of the cumbersome process of connection setup. Thanks to this benefit of PBC, it is increasingly adopted for smart control of CE and IT devices in the home and building environments.

**Figure 1:**
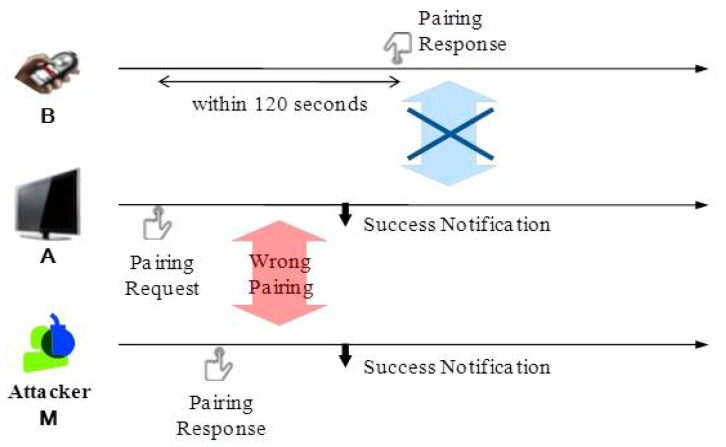
Attacker scenario.

However, one should be aware that there is a certain setup period (up to 2 min) to be allowed between pushing the first and second buttons so that a user can push a button of the first device and walk to the second device to push the second button. During that time, there is a possibility that unintended devices within range might break in by pressing its own button earlier than the second device as shown in Figure 1. An attacker may thus exploit this vulnerability to have his/her malicious device to be paired with the first device, which in turn yields eavesdropping of subsequent transactions or critical information. Moreover, the attacker may mount a Denial-of-Service (DoS) attack on the PBC service by having ongoing pairing processes constantly interfered. As such, PBC is never secure against malicious attackers located nearby.

With expanding the preliminary version of the paper [8], we present a Security-enhanced Push Button Configuration (SePBC) scheme in detail that significantly enhances the security of PBC by defeating the above-mentioned vulnerability of PBC. The key features behind SePBC are as follows:

- This paper proposes an efficient detection of the pairing establishment with a malicious or unintended device by bookkeeping all of the pairing messages during the setup period (of 2 min) without requiring the exchange of pre-shared keys a priori.
- The proposed approach identifies which pairing message is from a genuine or intended device by estimating the distances to all of the target devices (using ultrasound sensors) and deciding the closest as the pairing counterpart. Note that the proposed distance measurement protocol is not just about accuracy, but more about authenticity.
- Hence, this paper develops a secure handshake distance measurement protocol by preventing an adversary sitting outside the region from maliciously manipulating its distance.
- We actually verified the feasibility of the proposed mechanism by implementing RF and PWM-based ultrasound communication.

The SePBC scheme effectively realizes these ideas while causing no performance degradation in terms of pairing latency in the absence of attacks. Our security analysis results demonstrate its capability of defeating attacks on PBC providing high accuracy of attack detection. The rest of the paper is organized as follows. Section 2 overviews related works, and Section 3 identifies and formulates the problem. Section 4 presents the proposed approach in detail. Section 5 describes the details of implementation and the experimental results of the security analysis. Finally, the paper concludes with Section 6.

## 2. Related Works

### 2.1. Smart Home Control

Recently, the Internet of Things (IoTs) has been introduced, and hence, smart automation homes with IoT devices have further developed and become more popular in the real world. For example, smart home technologies have rapidly evolved, and hence, automatically controlled home equipment, such as TVs, lights, refrigerators and automatic windows, have appeared in the real-world market.

These smart automation homes’ IoT devices have been equipped with sensors and actuators to collect data and control devices. In the research area of such ubiquitous and embedded smart home control systems, many researchers including our group have devoted their efforts to developing power-efficient sensor networks and to support reliable and real-time communications between embedded sensor devices [9,10,11].

Incorporated with such underlying IoT networks, many various kinds of energy management systems have been proposed to enhance smart controls of the existing power grid [12,13,14,15,16]. These studies developed smart metering and control systems working together with smart IoT devices to reduce energy consumption, forecast energy requirements/conditions and satisfy consumers eventually. Especially, smart home case studies of Singapore reported challenges and limitations of current smart homes and IoT technologies [16]. Furthermore, one of the previous research works [17] was concerned about privacy issues of emerging smart energy metering systems and hence proposed a new privacy-enhanced smart metering method by applying a holomorphic encryption algorithm.

To make such smart home technologies practical and widely used in real-world households, security issues should be properly handled. However, compared to enterprise networks, smart home networks are very likely to be built without well-maintained centralized control and in-depth security technologies. This makes smart home platforms more vulnerable to malicious attempts. To address this issue, many research works have been presented recently [18,19,20,21,22,23,24]. First of all, several studies summarized and analyzed existing smart home devices and home networks with raising several security issues, such as compromising of outside servers, device auto-configuration, firmware update issues, and so on [18,19,20]. All of these studies pointed out that smart home technologies are dealing with personal life, finance and privacy; hence, it can be more attractive to attackers, and damage caused by such malicious attacks is very huge and irrevocable.

Furthermore, another previous work targeted smart home applications, such as SmartThing [3], Weave [2] and HomeKit [4], and examined the design flaws of these platforms from the programming point of view [21]. Especially, it focused on application programs developed in the Groovy environment [22]. Other study targeted a secure network for smart home platforms and developed SH-SecNet (Secure Networks for Smart Homes), which is an enhanced secure network architecture using MCA (Multivariate Correlation Analysis) to identify malicious and abnormal network flows. Specifically, a defensive platform has been proposed to block false data injection attacks on a smart grid network control center by developing a cumulative sum algorithm to quickly detect attacks [23] .

With the literature survey including cutting edge technologies, we observe that there are not many research studies for discussing and presenting specific solutions for PBC security flaws. Motivated by this observation, this paper first thoroughly examines security challenges in the current PBC mode of Wi-Fi in smart home environments and then proposes solutions.

### 2.2. Push Button Configuration

To make such smart home technology widely used, there has been a rapid increase in the need for the seamless and easy interplay of these embedded sensor devices, especially for user-friendly usage of consumer electronic devices. As a response to the need, Push Button Configuration (PBC) has been proposed to easily set up a secure session in both Wi-Fi WPS and RF4CE.

The PBC mode specified in Wi-Fi WPS [5] works as follows. After a user activates an AP and a device, a network name (SSID) for the AP is automatically generated and broadcast for discovery by the clients. Then, the user pushes buttons on both the AP and a CE device to initiate a security setup. This means the user is no longer involved in the complicated process of setting up a pass phrase, as security codes are activated automatically.

The PBC mode of ZigBee RF4CE is quite similar to that of Wi-Fi WPS in the sense that the user is capable of pairing two peer devices by pressing the buttons [6]. ZigBee RF4CE supports a push button pairing procedure for easy setting up of ZigBee IoT devices. Basically, Wi-Fi and ZigBee share the same concept of PBC, and hence, both protocols have a similar vulnerability problem that the original ZigBee pairing procedure and Wi-Fi PBC procedure are not capable of differentiating a legitimate pairing response from malicious or unintended attacks. Specifically, attackers in the PBC setting can passively deny the pairing process between devices or actively intercepting the secure session.

Bluetooth SSP (Simple Pairing Protocol) also supports a simple pairing protocol with four models [7] as follows.

- Numeric comparison
- Passkey entry
- Out of band
- Just works

The first and second modes assume at least one IoT device has display capability. With the *numeric comparison* mode, a user is supposed to compare two displays on both devices and push buttons when the two numbers are the same. With the *passkey entry* method, the user needs to enter a randomly-generated passkey with a keyboard or something like that. The third mode, *out-of-band*, is used only when both devices have additional communication methods such as NFC (Near Field Communication). The *just works* mode, numeric number comparison is internally performed without the user’s input. However, Just Works mode is vulnerable to malicious or unintended interrupt of communications. To protect the Bluetooth SSP against attackers, especially with the forth mode, we need a way to identify if or not pairing response is coming from a legitimate device or a malicious one. Note that this paper chooses a Wi-Fi protocol for our test platform and applies the proposed method for Wi-Fi because the Wi-Fi protocol is the most common and widely used in households. Although the proposed scheme is implemented for Wi-Fi networks in this paper, the main idea of this paper can be modified and extended for other MAC protocols including ZigBee and Bluetooth.

Although security issues of smart home technologies are actively studied, most of them have focused on general security issues and solutions from the applications’ and networks’ perspectives. On the other hand, there has been little effort specific to safeguard PBC against malicious attacks. Since PBC in WiFi-enabled devices plays an important role for easy interplay between IoT devices, it is critical to provide a high level of security for PBC. Motivated by this observation, this paper proposes a novel platform called SePBC and implements it for its feasibility verification.

To achieve the high level of security for PBC, the proposed method adopts ranging techniques [25,26,27] that aim to estimate the distance between two devices. These distance estimation methods are generally based on Received Signal Strength (RSS), Time Difference of Arrival (TDOA) and Time of Arrival (TOA). Furthermore, Angle of Arrival (AOA), which is the relative angle between devices, can be used to estimate distance. Among them, the RSS-based distance estimation method has been widely used thanks to its simplicity. However, this method might become less accurate as the distance becomes longer. This problem is mainly because of non-uniform signal propagation features and interference effects. In order to reduce this estimation error, averaging and smoothing techniques have been applied [26,27].

However, these previous localization methods have focused on the accuracy of locations, not the truth or falsehood of the calculated locations. These previous works assumed that information received from the target would be legitimate, not adversarial. Inspired by this observation, this paper proposes a secure distance measurement with home environments.

## 3. Problem Statement

In what follows, we identify a problem and present design goals to solve the identified problem.

### 3.1. Attack Scenario

We identified that the original PBC has a serious security vulnerability to cause a device to be paired with the wrong (malicious) device, possibly under the control of an attacker. This may happen if an attacker’s device (say **M**) is within the communication range of the devices (say **A** and **B**) executing the PBC pairing process. Figure 1 depicts one of the attack scenarios. If **M** presses a PBC button (to send a pairing response) prior to **B** as soon as **A** makes a pairing request, **A** sets up a secure session with **M** instead of **B**, while denying subsequent pairing response from **B**. This has the effect of disclosing all of the secure information to the attacker and/or mounting Denial-of-Service (DoS) attacks against legitimate devices. Note that this is a well-known open problem existing in both RF4CE and WiFi WPS [5].

### 3.2. Design Goals

We aim to design a defense mechanism, called Security-enhanced PBC (SePBC), to defeat the aforementioned attacks on PBC satisfying the following requirements.

- Compatibility with the original PBC: It is desirable to keep the PBC-based User Interface (UI) rather than introducing a brand new mechanism, and hence, it is critically important to make SePBC compatible with the original PBC in the sense that a user still presses buttons on devices without worrying about possible attacks. Accordingly, we use PairingRequest and PairingResponse messages defined in the original PBC as is.
- Accuracy of attack detection: SePBC must achieve highly accurate detection of attacks in terms of minimizing both false positives and false negatives.
- Low-cost: SePBC must use lightweight operations and employ inexpensive hardwares . Moreover, it needs to incur little overhead when there is no attack.

## 4. Proposed Approach

In what follows, we describe our key idea and the details of the proposed approach.

### 4.1. Key Idea

To develop our proposed scheme, we first raise two questions to be addressed as follows.

- Q1. How does one detect if the wrong pairing could have been made with a malicious or misbehaving device?
- Q2. How does one tell which pairing message is from the intended device and thus has to be accepted?

Q1 is related to the attack scenario of Figure 1, in which **A** receives multiple PairingResponse messages from **M** and **B** within the PBC timeout period of, say, 2 min. Therefore, **A** can decide, as soon as receiving the second pairing message, that something is wrong with the PBC pairing, thus requiring further inspection of the pairing messages for their genuineness. Q2 can be addressed by using the fact that a malicious device, **M**, would normally be located farther away from **A** than a benign device, **B**, because **M** has to be out of sight of **A** and/or separated by a barrier or wall to hide itself from **A**. To utilize this nature, one may consider an approach for **A** to reduce the power in transmitting the PairingRequest message. However, this is an incomplete solution in that an attacker may (1) still guess when the message was sent by sensing the channel (since a sensing range is much wider than the communication range) and (2) inject PairingResponse messages in response to the guessed PairingRequest. Of course, a brute-force, repeated injection would work, as well. We, therefore, take an approach to estimate the distance between **A** and **B/M** either directly or indirectly, as discussed below.

### 4.2. Assumption

**Figure 2:**
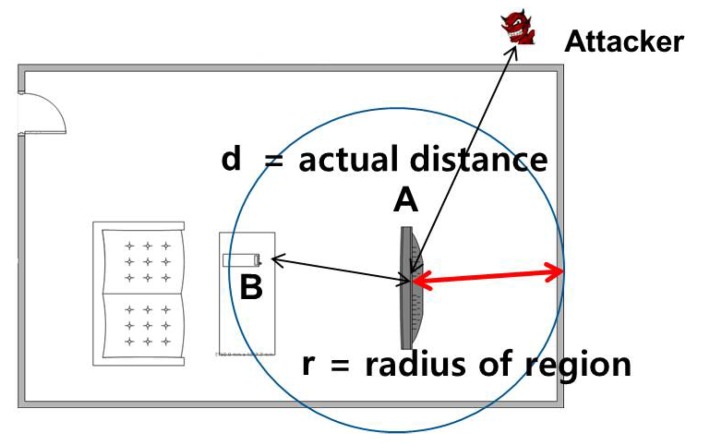
Attacker location and pre-defined region.

We make the following assumptions. First, device **A** is assumed to be trusted, while devices **B** and **M** are not. Second, all devices are capable of Radio Frequency (RF), as well as sound (or ultrasound) frequency communications, where the latter is an optional element. Third, each device has a known, bounded processing delay. Finally, we assume that legitimate devices are within a certain range of distance, as shown in Figure 2. In other words, if the actual distance, *d*, is larger than a pre-defined region, *r*, (i.e., d>r), we consider the device not to be legitimate.

### 4.3. How Does SePBC Work?

**Figure 3:**
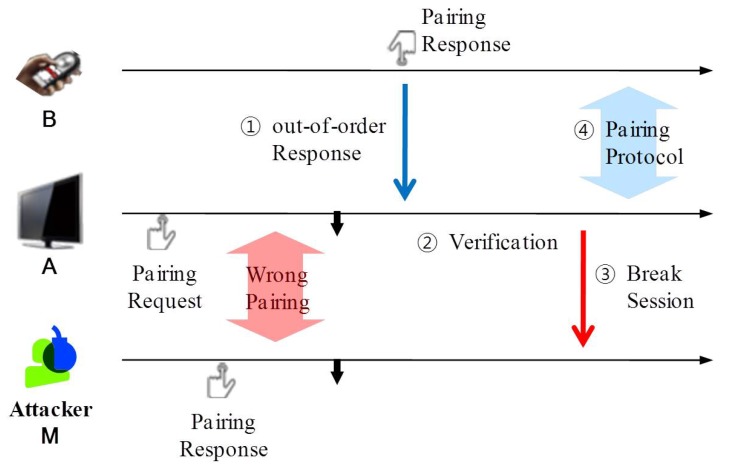
Key idea of the proposed approach.

Figure 3 illustrates how SePBC works under the attack scenario, in which a malicious **M** responds to PairingRequest from **A** earlier than **B**.

In the beginning, SePBC lets **A** make a pairing with the first device replying with PairingResponse, which is **M**. This way, SePBC incurs no additional latency in the PBC pairing when there is no attack. **A** then keeps on monitoring the arrival of PairingResponse messages from other devices. Hence, if **B**’s button is pressed, **A** receives an out-of-order PairingResponse, thus entering into “a verification stage” to determine which of the pairing responses is genuine. The verification scheme can be either or both of the RSS-based ( Received Signal Strength-based) and RTT-based (Round Trip Time based) schemes, which are described in the following section. If the verification result indicates A has been paired with the wrong device, it breaks the existing session with **M** and re-starts the PBC pairing protocol with **B**.

Each device should take two roles in parallel. That is, it serves both as a SePBC initiator controlling the entire process and as an SePBC counterpart responding to the initiator’s request. The former is activated when a user presses the device’s button first, while the latter when PairingRequest is received (due to the user’s pressing the other’s button).

### 4.4. Verification Scheme

To verify whether or not a certain target device is malicious, we present two methods, i.e., RSS- and RTT-based methods, as follows.

#### 4.4.1. RSS-Based Verification

Since **A** observes that the signal received from **M** is much weaker than that from **B**, we may compare the RSS values of PairingResponse messages and declare the strongest as the intended target because it is the closest to **A**. Alternatively, we may apply RSS-to-distance conversion to compute distances to **A** and decide which of the devices are within the user’s space. Clearly, the reason for RSS-based verification to work well in home/building environments is that barriers or walls significantly attenuate the RSS of messages of **M**, if the transmitted signal strength values are at the same level.

The RSS-based method is simple, but effective against PBC attacks in practical environments and, hence, can be used as a first line of defense that stresses the attacker to get closer to the victim or significantly increase the transmission power, taking the risk of getting caught.

#### 4.4.2. RTT-Based Verification

In certain applications requiring highly accurate detection of attacks, RSS may fail to meet the requirement due to its inherent estimation errors. To deal with this situation, we propose a second method that applies an RTT-based ranging technique. This RTT-based method attempts to accurately verify if a target device is located in a pre-defined region. In particular, device **A** wants to verify if each target device, **B** or **M**, is present, for example, in a living room. To describe the RTT-based method, we define *s* the speed of sound or 331 (m/s), *c* the speed of light or $3\times 10^{8}$ (m/s) and *r* the radius of the region in which the target device resides. We additionally define the following messages: RTT_Challenge(N) and RTT_Response(N), where N is a random number.

**Figure 4:**
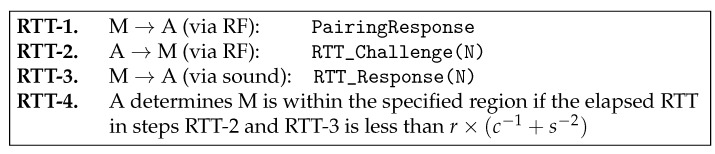
RTT-based distance verification protocol.

That said, **A** verifies **M** according to the proposed protocol in Figure 4, which is triggered by **A**’s reception of the PairingResponse (in RTT-1). **A** then sends RTT_Challenge(N) with random N via the RF interface (in RTT-2) and gets back, from **M**, RTT_Response(N) with the same N via the sound interface (in RTT-3). Finally, **A** checks the following condition.

(1) $$\begin{array}{c} RTT\leq r\times (c^{-1}+s^{-1}) \end{array}$$

Likewise, **A** can verify **B**. We now explain how this protocol works. When *d* refers to the actual distance between **A** and **M**, the time it takes in steps RTT-2 and RTT-3 are d/c and d/s, respectively. Hence, the total elapsed time should be $d\times (c^{-1}+s^{-1})$, which must be less than $r\times (c^{-1}+s^{-1}$) if **M** is within the region.

### 4.5. Security Challenges

In the design of the proposed verification scheme, there are several important security challenges we should address. We describe the challenges and introduce how we handle them in the proposed solutions.

**Figure 5:**
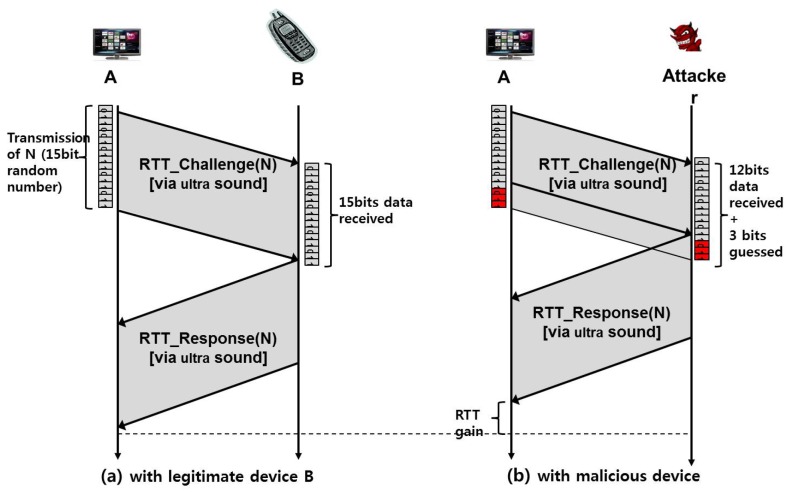
Potential security issue in ultrasound transmission.

**Figure 6:**
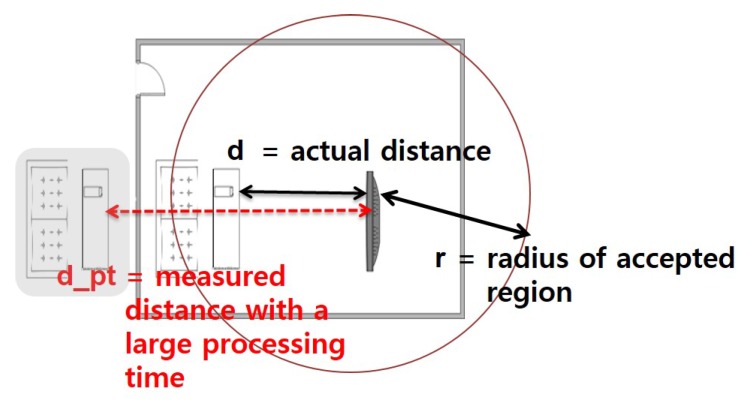
False alarm due to a large processing time.

- First, we use a sound (or ultrasound) interface for RTT-3 in Figure 4 instead of using RF communication, mainly because increased RTT measurements by the sound interface makes it less sensitive to the timing jitters, thus increasing the accuracy of verification.
- Second, the two proposed messages, RTT_Challenge(N) and RTT_Response(N), include a random number N. Thanks to this feature, an adversary sitting outside the region cannot fool the verifying node into thinking him/her to be inside, for the following reason. The adversary is unable to get his/her RTT_Response packet to arrive in time at the verifying device due to the law of physics, and hence, he/she has no other way but to start transmitting the packet earlier. However, this is impossible, as well, because the verifying device transmits a random number that cannot be guessed by the adversary and asks the same number to be returned, which means the adversary should wait for the completion of packet reception.
- Third, we use the sound interface only in a return path (**RTT-3:** RTT_Response(N)), not in a forwarding path (**RTT-2:** RTT_Challenge(N)). We intentionally design our protocol this way to prevent further potential attacks as described below. Suppose we use the sound interface in **RTT-2**, as well as in **RTT-3**. In this case, the attacking node **M** can return the signal in the middle of the transmission without waiting for the completion of packet reception. For example, in Figure 5, assuming that a random number N consists of 15 bits, a malicious node **M** can send RTT_Response(N) as soon as it receives the 12th bit without waiting for the last three bits, 13th~15th bits. By guessing the 13th~15th bits to be one of eight combinations (i.e., 000, 001, ..., 111), it can send a response message sooner than otherwise. In other words, with a 12.5% probability of success, an attacker **M** is able to return a “valid” RTT_Response(N) message without waiting for the completion of packet reception. By intentionally returning RTT_Response(N) earlier than when it was supposed to do, it can shorten the round-trip time. It can result in that the measured distance is shorter than the actual distance. Note that this issue is negligible in high-speed RF communication, but it can be problematic in low-speed ultrasound transmission. To protect the PBC against this potential attack, we send RTT_Challenge(N) via RF instead of sound transmission. On the other hand, in the return path (i.e., RTT_Response(N)), we can use sound transmission without worrying about this problem because we are sure that the destination of the RTT_Response(N) message is a legitimate device, not an attacker.
- Finally, there is another issue related to “processing time” at each device. Ideally, each device receives packets and sends them back immediately without any delay on the device. In this case, the round-trip time can be computed as the sum of inbound and outbound packet propagation and transmission times. However, in reality, each device requires some additional time duration to process the packet when it receives the packet before it sends it back. We call this delay the “processing time”. The processing time is not zero in practice, and furthermore, it is not a constant value, but depends on the platform specification of each device, such as CPU, memory, network card, and so on. As a consequence, some legitimate devices with large processing times might be mistakenly considered as attackers. Especially, general small size home appliances are likely to have low computing power CPUs and to generate large processing times. Figure 6 shows an example of such false alarms. In this figure, a remote control device, B, is physically located inside the acceptable range R. Suppose that the processing time of the device B is very large, and hence, its RTT measured at the server is too long. In this case, the device B might be considered as located outside the regions represented as the shaded area in Figure 6. To prevent this false alarm, this paper presents the extended version of the protocol, called eSePBC, in Section 4.6.

### 4.6. Handling of the Processing Time

**Figure 7:**
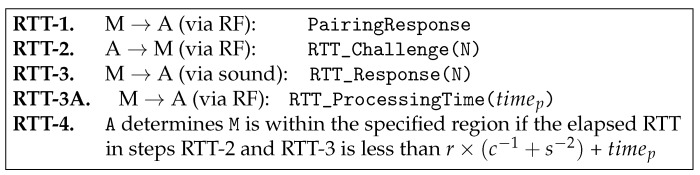
RTT-based distance verification protocol with considering the processing time, $time_{p}$, for each device.

Until now, we have assumed that the processing time at each device is either zero or negligible. In this subsection, we relax this assumption by considering low-power small home appliances with large processing times. Note that the processing time is not a constant value. The processing time depends on the platform specification of each device. To reflect such various processing time, we extend the proposed SePBC protocol in Figure 4 by adding the **RTT-3A** step as in Figure 7. Specifically, in **RTT-3A**, the remote target device sends its processing time information to the verifying device A. When A receives this message, it uses this value to properly compute the distance to the target device as shown in the following equation.

(2) $$\begin{array}{c} RTT-time_{p}\leq r\times \left(c^{-1}+s^{-1}\right) \end{array}$$

**Figure 8:**
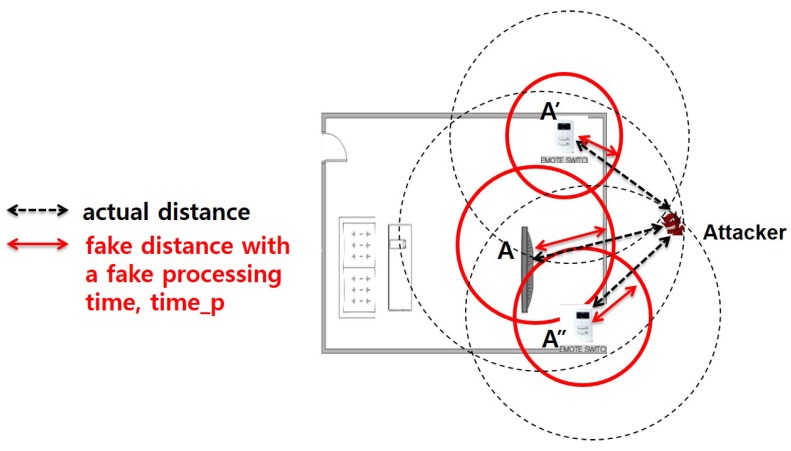
Further security protection.

However, this extension can cause critical security flaws. For example, the malicious device can send a large fake value of processing time instead of a true value. In this case, the attacker can fool the verifying node to consider that the malicious node is within the accepted region. To prevent this security problem, we now propose a cooperative security protection mechanism. When the verifying node A receives a $time_{p}$ value from the target device, it first checks whether or not the $time_{p}$ is larger than a threshold, $time_{thresh}$. If so, we can think that the processing time is way too large, and hence, the target device is suspicious. To verify the suspicious target device, we perform further investigation using a cooperative mechanism as illustrated in Figure 8. As shown in this figure, we deploy two or more security devices (e.g., nodes A’ and A”) and obtain measured distance information from these devices. If the processing time information is not fake, then the measured distances from three different points represented as a dotted line would generate a common area, which is the potential location of the target device. If the processing time information is fake and too large, represented as red solid lines in the figure, then three measured distances would not make any common intersection area, which does not make any sense at all. We formally model this idea in the following equations.

(3) $$\begin{array}{ccc} d_{A} & = & \left(RTT_{A}-t_{p}\right)*\left(c+s\right) \end{array}$$

(4) $$\begin{array}{ccc} d_{A^{\prime }} & = & \left(RTT_{A^{\prime }}-t_{p}\right)*\left(c+s\right) \end{array}$$

(5) $$\begin{array}{ccc} d_{A^{\prime \prime }} & = & \left(RTT_{A^{\prime \prime }}-t_{p}\right)*\left(c+s\right) \end{array}$$

(6) $$\begin{array}{ccc} Region_{A} & = & a\;\mathrm{Circle}\;\mathrm{with}\;a\;\mathrm{radius}\;R_{A}=d_{A}+\alpha \end{array}$$

(7) $$\begin{array}{ccc} Region_{A^{\prime }} & = & a\;\mathrm{Circle}\;\mathrm{with}\;a\;\mathrm{radius}\;R_{A^{\prime }}=d_{A^{\prime }}+\alpha \end{array}$$

(8) $$\begin{array}{ccc} Region_{A^{\prime \prime }} & = & a\;\mathrm{Circle}\;\mathrm{with}\;a\;\mathrm{radius}\;R_{A"}=d_{A^{\prime \prime }}+\alpha \end{array}$$

(9) $$\begin{array}{ccc} M\;\mathrm{is}\;\mathrm{an}\;\mathrm{attacker} & if & Region_{A}\cap Region_{A^{\prime }}\cap Region_{A^{\prime \prime }}==\emptyset \end{array}$$

where α is an extra value to accommodate ultrasound noises.

## 5. Implementation and Experimental Results

To validate the proposed scheme, we performed several experiments as follows.

### 5.1. Implementation

#### 5.1.1. Block Diagram

**Figure 9:**
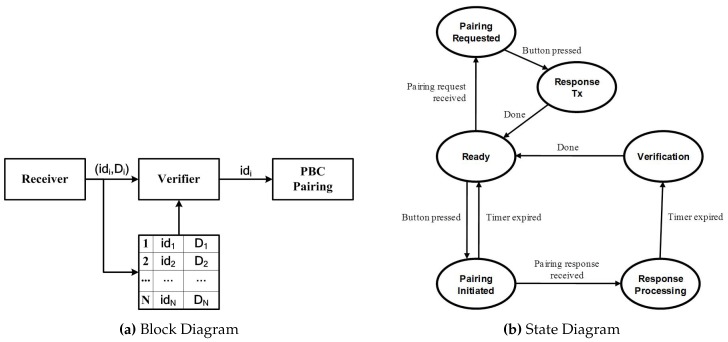
Implementation of SePBC.

Figure 9a presents the block diagram of SePBC, consisting of three building blocks, i.e., a receiver, a verifier and a PBC pairing module. It also uses a memory shared between the receiver and the verifier to store the history of the received pairing responses during the monitoring window. The *i*-th entry of this memory corresponds to the *i*-th response and a tuple, $\left(id_{i},D_{i}\right)$, where $id_{i}$ is the ID of the device having sent the pairing response and $D_{i}$ is the side information for verification, such as the RSS and/or RTT of the message from device $id_{i}$. Note that the value of $D_{i}$ is not conveyed in the received message, but extracted from the message (RSS-based) or estimated using the protocol in Figure 4 (RTT-based).

The operations of these building blocks are as follows. First, during the timeout interval starting from the transmission of the pairing request, the receiver:

1. accepts PairingRequest or PairingResponse messages from external devices via an RF interface;
2. constructs $D_{i}$ and combines it with the device’s id to form $\left(id_{i},D_{i}\right)$; and
3. writes this tuple into the memory, as well as informs the verifier of the arrival of the first message.

Second, the verifier:

1. upon receiving notification of the first message arrival, controls the PBC pairing module to set up a secure session with a device, $id_{1}$;
2. upon expiration of the timer, verifies, using RSS- and/or RTT-based schemes, each and every PairingResponse message to uncover harmful ones using the information stored in the memory; and
3. if the verification results indicate that the pairing in Step (1) is wrong, it breaks the existing session with $id_{1}$ and executes the pairing protocol with $id_{c}$, which has been determined to be the correct device.

Finally, the PBC pairing module is in charge of executing the PBC pairing protocol with the other device, either $id_{1}$ or $id_{c}$.

#### 5.1.2. State Transition Diagram

A state transition diagram for SePBC is shown in Figure 9b. It consists of six states, namely, **Ready** , **Pairing Requested**, **Response Sent**, **Pairing Initiated**, **Response Processing** and **Verification**. The SePBC operations of a device **A** at each of the states are as follows.

- **Ready** state: **A** waits for external events to trigger the SePBC; **A** makes a transition to the **Pairing Requested** state if receiving the PairingRequest message and to the **Pairing Initiated** state if its own PBC button is pressed.
- **Pairing Requested** state: **A** asks a user to press the PBC button and moves to the **Response Sent** state.
- **Response Sent** state: **A** returns to the **Ready** state after completing the transmission of the PairingResponse message.
- **Pairing Initiated** state: **A** first sends a PairingRequest message within the transmission radius and then waits for the reception of PairingResponse. **A** makes a transition to the **Response Processing** state if receiving the message within a setup interval, while going back to the **Ready** state if the setup timer expires with no message arrival. The latter means that no pairing takes place because it did not hear from any device.
- **Response Processing** state: **A** executes the pairing process with the first device, $id_{1}$, that has responded to the request. **A** then waits until the setup timer expires, receiving and storing the PairingResponse messages from other devices. Finally, **A** goes to the **Verification** state after the timer runs out.
- **Verification** state: if no other PairingResponse message was received, **A** concludes that the current pairing is normal and thus returns to the **Ready** state. By contrast, if there were multiple receptions of PairingResponse messages, **A** determines the target device to be paired with, using $D_{i}$’s in the memory. That is, in the case of using RSS values as $D_{i}$’s, it chooses the device $id_{c}$ that has the largest RSS value among others. Hence, if $id_{c}=id_{1}$, it again declares the current pairing as normal, taking no further action; otherwise, it breaks an existing pairing with $id_{1}$ followed by executing the pairing process with $id_{c}$. Finally, **A** makes a transition to the **Ready** state.

### 5.2. Experiment

**Figure 10:**
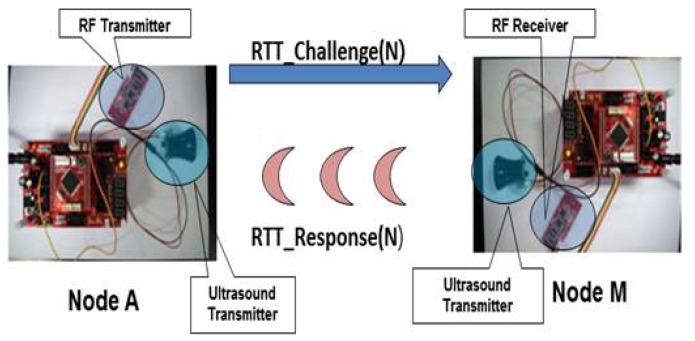
Experiment setup with Atmega128, RF and ultrasound modules.

**Figure 11:**
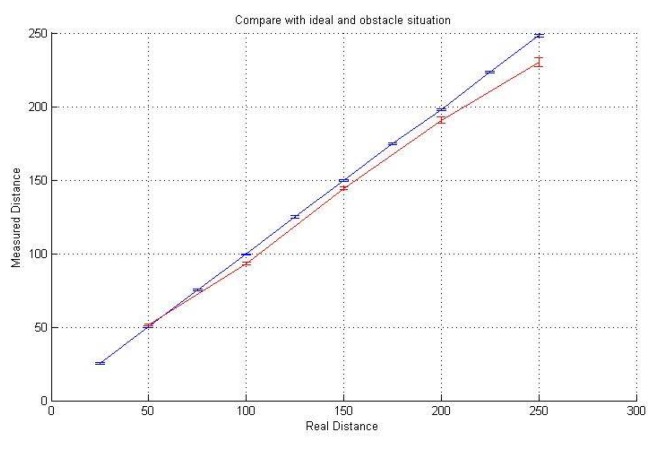
Real distance vs. measured distance (one-way).

To validate the proposed SePBC scheme, we performed several experiments using a ATmega128 board, AM-RFMOD (RF modules) and HG-M40RNII/HG-M40TNII (ultrasound modules), as shown in Figure 10. As a baseline, we first tested the feasibility of ultrasound-based distance measurement. As in Figure 11, we got the result that even in a home environment with obstacles, the measured distance is very similar to the actual distance.

Followed by the above baseline feasibility tests, we implemented the verification procedure described in the previous section. First, we developed the PWM-based transmission protocol to implement ultrasound-based data communication. In PWM (Pulse-Width Modulation), the widths of the pulses correspond to specific data values encoded at one end and decoded at the other [28]. In our implementation, we used nine kinds of purses, each of which has its own width, one of 24 ms, 40 ms, 56 ms, ..., 184 ms, as shown in Table 1. Each purse is mapped to one of the three-bit data, except that the longest purse corresponds to the flag as a boundary. In the current experiment setup, we transmitted four signals: one signal is used as a flag, and the other three signals compose a nine-bit random number N.

In the first set of experiments, we exchanged RTT_challenge(N) and RTT_Response(N) between two devices and measured an end-to-end round-trip time. Specifically, we set up experiments so that a source device **A** transmits RF signal with a nine-bit random number N and records the time gap between the time the RF signal was sent and the time the first ultrasound signal was received from the destination device. The measured round trip time is presented in Column (a) of Table 2. The measured round trip time includes (1) the transmission time of RTT_challenge(N) via RF from a source device to the destination, (2) the RF propagation delay of this RTT_challenge(N) message, (3) the processing time at the destination, (4) the transmission time of RTT_Response(N) via ultrasound from the destination to the source and (5) ultrasound propagation delay of this RTT_Response(N) message. In this case, the RF-related first two time factors are very small and negligible. Hence, we dissect the measured end-to-end RTT focusing on the other three factors: the processing time and ultrasound-related time factors.

Next, we measured the processing time at the destination device as presented in Column (b). Specifically, we measured the gap between the time the destination device receives the data via the RF module and the time it sends back the first purse to a source via the ultrasound module. We repeated the measurements with varying the value of N from 000000000 to 111111111 and also varying the distance between two devices, as well. Not surprisingly, we observed that the processing time measured on the current ATmega128 board used in this experiment is constant (i.e., 2.35 ms), regardless of the value of N and the distance between devices. Note that 2.35 ms in ultrasound transmission is correspondent to about 0.8 m. This implies that even with the use of the highest performance device, an attacker can manipulate its location by at most 0.8 m.

Then, we compute transmission time for each data N sent via RTT_Response(N) by adding up the widths of one flag (= 84 ms) and three data signals. For example, when N = 001001001, the transmission time of RTT_Response(N) is computed as 184 ms (flag) + 40 ms (001) + 40 ms (001) + 40 ms (001). We also calculate the theoretical propagation delay of ultrasound transmission based on the distance between two devices. Assuming the medium is air, we divided the distance by 340 m/s. Table 2 shows our experimental results with seven different values of N when distance = 6 m. From the results, we observed that the potential measurement noise is less than 2.5 ms, which corresponds to 0.85 m. Considering the PBC is usually deployed for personal area communication between consumer devices in a home environment and an attacker is likely to be outside the home, such measurement noise would be acceptable.

In the second set of experiments, we measured end-to-end round trip time with varying the distance from 1 m to 6 m between the source and destination devices. We repeat the above measurement 30 times for each distance. Table 3 shows the results with the minimum, median and maximum round trip time values for each distance among 30 results. From the results, we first observed that for every distance, the measured round trip time is very reliable in the sense that min, median and max values are not much different from each other. More importantly, we also found that the round trip time is linearly increasing as the distance increases. Specifically, the round trip time gap between each distance is less than 1.5 ms. As an example of 4.5 m and 5 m, the round trip time gap is 322.608 ms − 321.200 ms = 1.408 ms. This time gap multiplied by the speed of sound (340 m/s) is equal to 0.4872 m, which is very close to the actual distance gap between 4.5 m and 5 m.

In summary, we believe that the above results show the validity of the proposed distance-based verification procedure in SePBC with ultrasound and RF communication.

### 5.3. Discussion

The RSS-based verification is effective when there is a wall or a barrier separating a device from an adversary because it gives enough attenuation of the received signal to overcome the fluctuations in RSS measurements. Obviously, this method may fail if the adversary transmits its messages with maximum power, but doing so will greatly stress the adversary to consume a significant amount of energy. As such, it can be used as a first line of defense.

The RTT-based verification enables accurate detection of the adversary thanks to its capability to estimate the distance from the verifying device to the target device by counting the amount of RTT. This method is secure in the sense that the adversary sitting outside the region cannot fool the verifying node into thinking him/her to be inside, for the reasons as we presented in Section 4. The RTT-based verification can only determine if the target is within a circle, *C*, centered at the verifying device. By contrast, we are interested in testing the presence of the target in the rectangular or square region, *R* (such as a room). To deal with this dilemma, we may adjust the radius of *C* to make it fully contained in *R* and look for a target device that is within *C*. This has the effect of rejecting benign devices located at the corner of the room.

The proposed method aims to minimize additional overheads and modification of the existing PBC in Wi-Fi WPS while improving security at the same time. Note that the proposed SePBC does not require the proposed additional security-related procedure in most normal cases with no attack. It goes into such a verification stage only when it detects an abnormal neighbor device behavior. This implies that overheads of the proposed scheme do not affect the performance of Wi-Fi-based IoT devices in most cases. The overheads of the proposed scheme might occur due to bookkeeping of its own device and neighbor device information as described in Figure 9b. Furthermore, sending packets via ultrasound modules with PWM mode might cause overhead, as well. Overheads of the proposed methods for the ATmega128 board are summarized in Table 4 compared to one of the most common pass phrase-based methods with the SHA-1 encryption algorithm [29]. As shown in this table, the proposed SePBC can provide security even at low computational overhead at the device side mainly because it does not require complex cryptography calculation. Thanks to its simplicity, most of the processing time of SePBC is due to handling RF and ultrasound modules. In contrast, the pass phrase-based method using SHA-1 incorporates CPU-intensive calculation and, so, causes a longer processing time. Furthermore, we compared the code size, because some IoT devices, such as small home appliances, might have a memory limitation. The required code size of the proposed scheme is half of an SHA-1 module. This implies that the proposed SePBC can be more suitable for general small-sized home appliances, which are our target models in this paper.

## 6. Conclusions

As smart home IoT devices have recently emerged, privacy and security issues and solutions have been presented by many previous studies. However, there has been few efforts to specifically protect PBC against malicious attacks. Furthermore, most previous security solutions are supposed to be equipped with additional expensive hardware or complex encryption software. From this perspective, the proposed SePBC in this paper has several contributions. First, we identify security challenges and flaws of the current PBC mode of Wi-Fi protocols in detail. Based on that, we propose a practical solution to provide a high level of security for PBC while making the proposed SePBC protocol compatible with an original PBC in the sense that a user still presses buttons on devices without worrying about possible attacks. In addition, the proposed distance measurement scheme is more suitable for SePBC compared to previous localization algorithms because the proposed method focuses on identification of the truth or falsehood of location information rather than the detailed accuracy of the location itself. Specifically, we develop a secure handshake distance measurement protocol by preventing an adversary sitting outside the region from maliciously manipulating its distance to be fake. Finally, we have implemented and developed the proposed idea using ultrasound sensors to verify the feasibility of the proposed scheme in the real world. Thanks to the proposed SePBC, we believe that smart home IoT devices can make sessions with each other more securely and easily than before, and hence, more smart home applications can become available.

## Figures and Tables

**Table 1:** Encoding/decoding in the proposed PWM method.

Period (Pulse Width)	3-Bit Data Frame
24 ms	000
40 ms	001
56 ms	010
72 ms	011
88 ms	100
104 ms	101
120 ms	110
137 ms	111
184 ms	Boundary (flag)

**Table 2:** Measured round trip time (ms) of RTT_Challenge(N) and RTT_Response(N) messages when distance = 6 m.

	Measured End-to-End RTT	Processing Time	Transmission Time of RTT_Response(N) (c)		Propagation Delay (Distance = 6 m)	Noise (e = |a − (b + c + d)|)
N(9 Bit Random Number)	(a)	(b)	1 Purse (Frag Frame)	3 Purses (9 Bit Data Frame) (= Sum of 3 Frame Widths)	(d)	(e)
001001001	325.5	2.35	184	120	17.65	1.5
010010010	374.376	2.35	184	168	17.65	2.376
011011011	422.4	2.35	184	216	17.65	2.4
100100100	466.9	2.35	184	264	17.65	1.1
101101101	518.48	2.35	184	312	17.65	2.48
110110110	566.488	2.35	184	360	17.65	2.488
111111111	613.9	2.35	184	411	17.65	1.1

**Table 3:** Measured end-to-end round trip time.

Distance (cm)	Min (ms)	Median (ms)	Max (ms)
100	311.184	311.200	311.200
150	312.480	312.480	312.528
200	313.856	313.904	313.968
250	315.264	315.296	315.312
300	316.688	316.688	316.704
350	318.160	318.176	318.176
400	319.632	319.648	319.648
450	321.120	321.200	321.232
500	322.592	322.608	322.704
550	324.032	324.080	324.160
600	325.536	325.584	325.568

**Table 4:** Summary of the overheads of SePBC compared to the SHA-1-based algorithm for the ATmega128 board.

Method	Processing Time at a Device A	Code Size (bytes)
SePBC	2.7 ms	about 1500
SHA-1	4.9 ms	about 3900 (only cryptography module)

## Abbreviations

The following abbreviations are used in this manuscript:

PBC	Push Button Configuration
SePBC	Secure Push Button Configuration
TDOA	Time Difference of Arrival
RSS	Received Signal Strength
AOA	Angle of Arrival
